# Overexpression of WNT16 Does Not Prevent Cortical Bone Loss Due to Glucocorticoid Treatment in Mice

**DOI:** 10.1002/jbm4.10084

**Published:** 2018-10-23

**Authors:** Imranul Alam, Dana K Oakes, Austin M Reilly, Caylin Billingsley, Shahed Sbeta, Rita L Gerard‐O'Riley, Dena Acton, Amy Sato, Teresita Bellido, Michael J Econs

**Affiliations:** ^1^ Department of Medicine Indiana University School of Medicine Indianapolis IN USA; ^2^ Indiana Center for Musculoskeletal Health Indiana University School of Medicine Indianapolis IN USA; ^3^ Department of Anatomy and Cell Biology Indiana University School of Medicine Indianapolis IN USA; ^4^ Department of Medical and Molecular Genetics Indiana University School of Medicine Indianapolis IN USA

**Keywords:** WNT16, GLUCOCORTICOID, TRANSGENIC, BONE MASS, GENE, OSTEOPOROSIS

## Abstract

Glucocorticoids (GC) are commonly used for the treatment of a wide variety of autoimmune, pulmonary, gastrointestinal, and malignancy conditions. One of the devastating side effects of GC use is osteoporotic fractures, particularly in the spine and hip. Bisphosphonates (BP) are the most commonly prescribed pharmacological agents for the prevention and treatment of GC‐induced osteoporosis (GIO). However, GIO is marked by reduced bone formation and BP serves mainly to decrease bone resorption. The WNT signaling pathway plays a major role in bone and mineral homeostasis. Previously, we demonstrated that overexpression of *WNT16* in mice led to higher bone mineral density and improved bone microarchitecture and strength. We hypothesized that *WNT16* overexpression would prevent bone loss due to glucocorticoid treatment in mice. To test our hypothesis, we treated adult wild‐type and WNT16‐transgenic mice with vehicle and GC (prednisolone; 2.1 mg/kg body weight) via slow‐release pellets for 28 days. We measured bone mass and microarchitecture by dual‐energy X‐ray absorptiometry (DXA) and micro‐CT, and performed gene expression and serum biochemical analysis. We found that GC treatment compared with the vehicle significantly decreased femoral areal bone mineral density (aBMD), bone mineral content (BMC), and cortical bone area and thickness in both wild‐type and transgenic female mice. In contrast, the trabecular bone parameters at distal femur were not significantly changed by GC treatment in male and female mice for both genotypes. Further, we observed significantly lower level of serum P1NP and a tendency of higher level of serum TRAP in wild‐type and transgenic mice due to GC treatment in both sexes. Gene expression analysis showed lower mRNA levels of *Wnt16*, *Opg*, and *Opg/Rankl* ratio in GC‐treated female mice for both genotypes compared with the sex‐matched vehicle‐treated mice. These data suggest that although WNT16 overexpression resulted in higher baseline bone mineral density and bone volume per trabecular volume (BV/TV) in the transgenic mice, this was insufficient to prevent bone loss in mice due to glucocorticoid treatment. © 2018 The Authors *JBMR Plus* published by Wiley Periodicals, Inc. on behalf of American Society for Bone and Mineral Research.

## Introduction

Glucocorticoids (GC) are commonly used in patients for the treatment of a wide variety of autoimmune, pulmonary, and gastrointestinal conditions, as well as in post‐transplant and malignancy treatments. An estimated 1.2% of individuals ages 20 to 80 years in the US receive prescriptions for GC, with 28% of these patients using GC for more than 5 years.^1^ GC is also used frequently in other countries.^(2)^ One of the most ignored and devastating side effects of GC use is glucocorticoid‐induced osteoporosis (GIO), the most common form of secondary osteoporosis.^1^, ^3^ GIO has a significant risk (30% to 50%) of osteoporotic fractures, particularly in the hip and spine.^2^ Thus, GIO causes substantial morbidity and huge economic burden worldwide.

Previous studies demonstrated that GC administration has significant negative impact on skeletal health.^3^, ^4^, ^5^ GC decreases bone formation, increases bone resorption, as well as increases bone cell death.^4^, ^5^ Thus, GC treatment rapidly deteriorates bone mineral density (BMD) and strength, substantially increasing fracture risk in patients. Importantly, this fracture risk at a specific bone mineral density is greater for patients taking GC than their non‐GC counterparts; therefore, treatment to prevent osteoporosis is even more vital in patients taking GC.^6^ Currently, bisphosphonates (BP) are the most commonly used pharmacological agents for the prevention and treatment of GIO.^1^, ^4^ The benefits of BP are mostly attributable to their antiresorptive effects; however, the primary hallmark of GIO is reduced bone formation. Additionally, long‐term BP treatment has been associated with increased bone micro‐damage, osteonecrosis, and atypical fracture.^7^, ^8^ Parathyroid hormone (PTH) therapy such as teriparatide (recombinant human parathyroid hormone, analog 1‐34) and abaloparatide (an analog of human parathyroid related peptide, PTHrp 1‐34) enhance bone formation. However, both are contra‐indicated in children and patients with a history of skeletal malignancy or Paget's disease. Therefore, it is crucial to identify a different approach leading to more bone formation, in addition to prevention of bone loss, for the treatment of this condition.

The WNT (Wingless‐type mouse mammary tumor virus integration site) signaling pathway plays a major role in embryonic development and postnatal health and disease, including bone and mineral balance.^9^ The WNT family is composed of 19 secreted cysteine‐rich glycoproteins, and genomewide association studies demonstrated strong associations between WNT16 and both BMD and fracture risk across multiple populations.^10^, ^11^ In addition, studies involving mice revealed that WNT16 is a critical regulator for bone homeostasis.^12^, ^13^ One of the mechanisms by which GC suppress bone formation is through their effects on Wnt‐signaling pathway.^14^, ^15^ Recently, we created transgenic (TG) mice that overexpress human WNT16 in bone tissue and demonstrated that the TG mice displayed significantly higher bone mineral density in whole body, hip, and spine starting as early as age 6 weeks in both males and females.^13^ In addition, TG mice had up to 13‐fold higher bone volume at the inner trabecular bone and significantly higher bone area and thickness at the outer cortical bone, indicating that WNT16 overexpression in TG mice also improved bone microarchitecture. Higher bone density and superior bone quality in the TG mice led to higher bone strength in these mice. Mechanistically, WNT16 overexpression increased bone formation and reduced bone resorption in the TG mice.^13^ Furthermore, we detected significant reduction (55%) of Wnt16 mRNA expression in bone tissue in mice treated with GC. Together, these results suggest that enhancing WNT16 expression in bone tissue might have great potential to improve bone mass and strength in GIO. We hypothesized that *WNT16* overexpression would prevent bone loss and prevent bone fragility due to glucocorticoid treatment in mice. To test our hypothesis, we treated adult wild‐type and WNT16‐transgenic mice with vehicle and GC (prednisolone; 2.1 mg/kg body weight) via slow‐release pellets for 28 days. Our data suggest that although WNT16 overexpression in the transgenic mice resulted in higher baseline and final bone mass and improved bone microarchitecture, these mice still lost bone due to glucocorticoid treatment.

## Materials and Methods

### Generation of the Col2.3‐hWNT16 transgenic mice

The generation of the Col2.3‐hWNT16 transgenic mice was described previously.^13^ Briefly, the cDNA of human WNT16 was cloned into the pJ251 plasmid between the osteoblast‐specific 2.3 kb α1 type 1 collagen promoter and mouse protamine 1 polyA signal. The transgene expression construct (promoter + WNT16 cDNA + polyA tail sequence) was microinjected into pronuclei of B6 fertilized eggs, which were then transferred into C57BL/6 (B6) foster mothers by the Indiana University Institutional Transgenic Animal Facility. The integration of the transgene(s) into the genome of founder mice was determined by PCR using tail DNA.

### Experimental animals

We used 6 male and 6 female mice per genotype (Col2.3‐hWNT16 TG and wild‐type littermates as controls) in this study unless stated specifically otherwise. Adult transgenic and wild‐type control mice at 16 weeks of age were delivered placebo and prednisolone (2.1 mg/kg/d) (Innovative Research of America, Sarasota, FL, USA) via slow‐release pellets for 28 days under isoflurane anesthesia. To provide pain relief, mice were injected analgesic (Carprofen 10 mg/kg) subcutaneously approximately 1 hour before the pellet implantation. Food and water consumption, body condition and mobility, and pellet implantation sites were monitored regularly. All mice were generated and maintained at Indiana University. Mice were housed in polycarbonate cages in a vivarium maintained on a 12‐hour light and 12‐hour dark cycle and were fed a regular diet and water *ad libitum*. The procedures performed throughout the experiment were in accordance with the ethical standards and guidelines of the Indiana University Animal Care and Use committee (IACUC).

### Euthanasia and specimen collection

Mice were euthanized at 28 days after pellet implantation. The lower limbs (femur and tibia) and lumbar 3 through 5 vertebrae were dissected from these animals. The femora on the right side were immediately stored at −80 °C in saline‐soaked gauze for subsequent biomechanical testing. The femora on the left side were stripped of muscle, fixed in 10% neutral buffer formalin for 48 hours before transferred to 70% ethyl alcohol and stored at 4 °C for densitometry analyses. In addition, after removing of muscle and periosteum, both ends of the tibia and humeri were cut to flush out the marrow cavity with PBS before transferring them to RNA later stabilization reagent (Qiagen, Valencia, CA, USA). We also harvested muscles (gastrocnemius and quadriceps) from both placebo and prednisolone‐treated wild‐type and Col2.3‐hWNT16 transgenic mice, the wet weights of which were used for muscle weight analysis.

### Dual‐energy X‐ray absorptiometry (DXA)

The whole body, femur and lumbar vertebrae 1 through 5 of both the placebo and prednisolone‐treated wild‐type and Col2.3‐hWNT16 transgenic mice were scanned at 28 days after pellet implantation using DXA (PIXImus II mouse densitometer; Lunar Corp., Madison, WI, USA) with ultra‐high resolution (0.18 × 0.18 mm/pixel) as described previously.^13^ The global window for the whole body was defined as the whole body image minus calvarium, mandible, and teeth. After completion of the scan of each bone, mutually exclusive region of interest (ROI) boxes were drawn around the bone from which femur areal bone mineral density (aBMD; g/cm^2^) and bone mineral content (BMC; g) measurements were obtained. In addition, from the whole body scans, the percent fat (%) and total body mass (g) were measured. The lean body mass (g) was measured by subtracting the body fat mass from the total body mass and fat mass was normalized by the lean body mass.

### Micro‐computed tomography (µCT) analysis

The femurs and lumbar 5 vertebrae of both placebo and prednisolone‐treated wild‐type and Col2.3‐hWNT16 transgenic mice were scanned at 28 days after pellet implantation with a high‐resolution µCT scanner (vivaCT 40, Scanco Medical AG, Bruttisellen, Switzerland) with an isotropic voxel size of 10.5 µm^3^ as described previously.^13^ In brief, from the scout‐view, the growth plate location was identified and trabecular bone measurements consisting of 200 slices (2.1 mm) were completed from about 1 mm below the growth plate. Lumbar 5 vertebrae measurements included the vertebral body from the cephalad to the caudal endplate excluding the cortical bone. For cortical bone analysis, the mid‐femur of each bone was determined from the scout‐view and a total of 60 slices (30 slices above and 30 slices below the mid‐femur) were scanned with the same setting as described previously.^13^ Finally, 3D and 2D morphometric evaluations were performed for the cortical and trabecular bone from each scan, and bone volume (BV/TV) and structural parameters (trabecular number [Tb.N], trabecular thickness [Tb.Th], trabecular separation [Tb.Sp], cortical bone area [B.Ar/T.Ar], cortical thickness [Ct.Th]) were determined. The use of the nomenclature, symbols, and units were followed as described in Bouxsein and colleagues.^16^

### Biomechanical measurements

Three‐point bending measurement of whole‐bone strength of femur of both placebo and prednisolone‐treated wild‐type and Col2.3‐hWNT16 transgenic mice at 28 days after pellet implantation was performed using an electromechanical test machine (TestResources, Shakopee, MN, USA) as described previously.^13^ In brief, the femurs were held in place by small preload (<1 N) and each bone was loaded to failure in monotonic compression using a crosshead speed of 0.2 mm/s, during which force and displacement measurements were collected every 0.02 s. From the force versus displacement curves, stiffness (N/mm), ultimate force (N), energy to ultimate force (mJ), and energy to failure (mJ) were calculated using MTestWR software following standard equations.

### Serum biomarkers

Serum levels of mouse pro‐collagen 1 intact N‐terminal (P1NP), carboxy‐terminal collagen cross‐links (CTX) and tartrate‐resistant acid phosphatase 5, isoform b (TRAcP5b) of both placebo and prednisolone‐treated wild‐type and Col2.3‐hWNT16 transgenic mice at 28 days after pellet implantation were measured by ELISA kits (Immunodiagnostic Systems, Scottsdale, AZ, USA, and Biomedical Technologies Inc., Stoughton, MA, USA) according to the manufacturers’ instructions. As our previous study^14^ demonstrated, changes of serum levels of P1NP occur as early as two weeks post‐GC treatment, we also measured this value at 2 weeks after pellet implantation in GC‐treated wild‐type and transgenic mice. Serum calcium (Ca), phosphorus (Phos), blood urea nitrogen (BUN), creatinine (CREA), and alkaline phosphatase (ALP) were measured using the Randox Rx kit (Daytona Analyzer, Randox Laboratories, Charles Town, WV, USA).

### Gene expression analysis

Measurements of gene expression were performed by real‐time PCR using bone tissue (femur) from both placebo and prednisolone‐treated wild‐type and Col2.3‐hWNT16 transgenic mice at 28 days after pellet implantation as described previously.^13^ All qPCR reactions were performed using the custom‐made primer and probe sets from Integrated DNA Technology (IDT, Coralville, IA, USA) or from Applied Biosystems (Carlsbad, CA, USA) for human *WNT16* (*hWNT16*), endogenous mouse *Wnt16* (*Wnt16*), runt‐related transcription factor 2 (*Runx2*), alkaline phosphatase (*Alp*), osteocalcin (*OC*), osteoprotegerin (*Opg*), and tumor necrosis factor (ligand) superfamily, member 11 (*Rankl* or *Tnfs11*). Real‐time detection of PCR products was accomplished using an ABI PRISM 7900 sequence detector (Applied Biosystems) and normalized to the housekeeping gene beta‐Actin.

### Statistical analysis

Quantitative data were expressed as mean ± SEM. Statistical differences between placebo and prednisolone‐treated groups were tested in wild‐type and Col2.3‐hWNT16 transgenic mice using the unpaired Student's *t* test. In addition, a two‐factor ANOVA analysis was performed to identify the interaction effects of GC treatment with genotype for all measured parameters. All statistical analysis was performed using the statistical software package StatView (Abacus Concepts, Inc., Berkeley, CA, USA). The level of significance was set at *p* ≤ 0.05.

## Results

### GC treatment significantly lowered the body weight in both wild‐type and Col2.3‐hWNT16 TG mice

**Figure 1:**
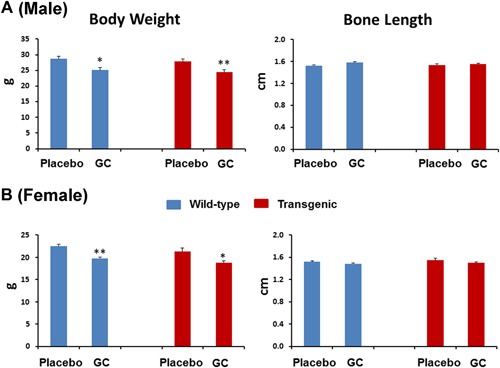
Measurement of body weight and bone length. The body weight measured at 28 days after pellet implantation showed that both male (*A*) and female (*B*) wild‐type mice had significantly lower body weight due to GC treatment compared with the placebo‐treated control mice. The Col2.3‐hWNT16 TG mice treated with GC also displayed significantly lower body weight in both male and female mice compared with the placebo group. Both male and female wild‐type mice treated with placebo and GC showed similar femur length at 28 days after pellet implantation. In addition, bone length in Col2.3‐hWNT16 TG mice treated with placebo and GC did not differ in both sexes. Values are given as mean ± SEM; *p < 0.05 and **p < 0.005 versus genotype‐matched placebo‐treated mice using Student's *t* test. GC = glucocorticoid.

The baseline body weight demonstrated no significant difference between placebo and GC‐treated male and female wild‐type and transgenic mice (Supplemental Table S1). The body weight measured at 28 days after pellet implantation showed that both male and female wild‐type mice had significantly lower body weight (*p* < 0.05 and *p* < 0.005, respectively) due to GC treatment compared with the placebo‐treated control mice (Fig. 1 *A*, *B*). The Col2.3‐hWNT16 TG mice treated with GC also displayed significantly lower body weight in both male (*p* < 0.005) and female (*p* < 0.05) mice compared with the placebo group (Fig. 1 *A*, *B*).

### Bone lengths are similar in GC‐treated wild‐type and Col2.3‐hWNT16 TG mice compared with the placebo‐treated mice in both male and female

Both male and female wild‐type mice treated with placebo and GC showed similar femur length at 28 days after pellet implantation (Fig. 1 *A*, *B*). In addition, bone length in Col2.3‐hWNT16 TG mice treated with placebo and GC did not differ in both sexes, suggesting that GC has minimum effect on growth of adult bone (Fig. 1 *A*, *B*). Also, two‐way ANOVA analysis demonstrated no significant interaction effects of GC treatment with genotype for bone length (Supplemental Table S2).

### Areal BMD and BMC of whole body and spine are similar in GC‐treated wild‐type and Col2.3‐hWNT16 TG mice compared with the placebo‐treated mice in both male and female

The baseline whole body and spine aBMD and BMC were similar between placebo and GC‐treated male and female wild‐type and transgenic mice (Supplemental Table S1). Male and female Col2.3‐hWNT16 TG mice treated with placebo had significantly higher whole body aBMD (*p* < 0.006 and *p* < 0.0001, respectively) and BMC (*p* < 0.01 and *p* < 0.01, respectively) compared with WT placebo group at 28 days after pellet implantation (Supplemental Fig. S1*A*, *B*). GC treatment did not change whole body aBMD significantly in male and female wild‐type and Col2.3‐hWNT16 TG mice compared with placebo‐treated mice. A slight but nonsignificant decrease of whole body BMC was observed in GC‐treated male and female mice in both genotypes except male TG mice treated with GC displayed significantly lower BMC compared with sex‐matched placebo group (Supplemental Fig. S1*A*, *B*). Male and female Col2.3‐hWNT16 TG mice treated with placebo had significantly higher spine (L_1–5_) aBMD (*p* < 0.02 and *p* < 0.001, respectively) and BMC (*p* < 0.02 and *p* < 0.01, respectively) compared with WT placebo group (Supplemental Fig. S2*A*, *B*). GC treatment did not significantly change spine aBMD and BMC in male and female wild‐type and Col2.3‐hWNT16 TG mice compared with placebo‐treated mice (Supplemental Fig. S2*A*, *B*). Two‐way ANOVA analysis demonstrated no significant interaction effects of GC treatment with genotype for whole body and spine aBMD and BMC (Supplemental Table S2).

### GC treatment significantly lowered femoral aBMD and BMC in both wild‐type and Col2.3‐hWNT16 TG female mice

**Figure 2:**
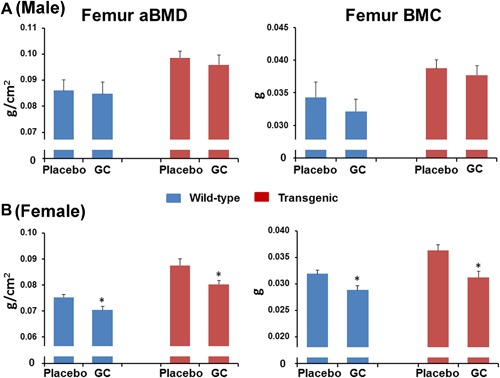
Femur aBMD and BMC measured by DXA. Male (*A*) and female (*B*) Col2.3‐hWNT16 TG mice treated with placebo had significantly higher femoral aBMD and BMC compared with WT placebo group at 28 days after pellet implantation. Female wild‐type mice treated with GC had significantly lower femoral aBMD and BMC compared with the placebo‐treated mice. Similarly, female Col2.3‐hWNT16 TG mice treated with GC had significantly lower femoral aBMD and BMC compared with the placebo‐treated mice. Values are given as mean ± SEM; *p < 0.05 and **p < 0.005 versus genotype‐matched placebo‐treated mice using Student's *t* test. GC = glucocorticoid.

The baseline femur aBMD and BMC showed no significant difference between placebo and GC‐treated male and female wild‐type and transgenic mice (Supplemental Table S1). Male and female Col2.3‐hWNT16 TG mice treated with placebo had significantly higher femoral aBMD (*p* < 0.03 and *p* < 0.002, respectively) and BMC (*p* = 0.01 and *p* < 0.01, respectively) compared with WT placebo group at 28 days after pellet implantation (Fig. 2 *A*, *B*). Male wild‐type mice treated with GC had slightly but nonsignificantly lower femoral BMC compared with the placebo‐treated mice (Fig. 2 *A*). Female wild‐type mice treated with GC had significantly lower femoral aBMD and BMC (*p* < 0.05 and *p* < 0.05, respectively) compared with the placebo‐treated mice (Fig. 2 *B*). Similarly, female Col2.3‐hWNT16 TG mice treated with GC had significantly lower femoral aBMD and BMC (*p* < 0.05 and *p* < 0.05, respectively) compared with the placebo‐treated mice (Fig. 2 *B*). Two‐way ANOVA analysis demonstrated no significant interaction effects of GC treatment with genotype for femur aBMD and BMC (Supplemental Table S2).These data suggest that overexpression of WNT16, although increasing bone mass overall, did not prevent bone loss due to GC treatment in the female transgenic mice.

### GC treatment did not change bone microarchitecture in cancellous bone in distal femur measured by μCT

**Figure 3:**
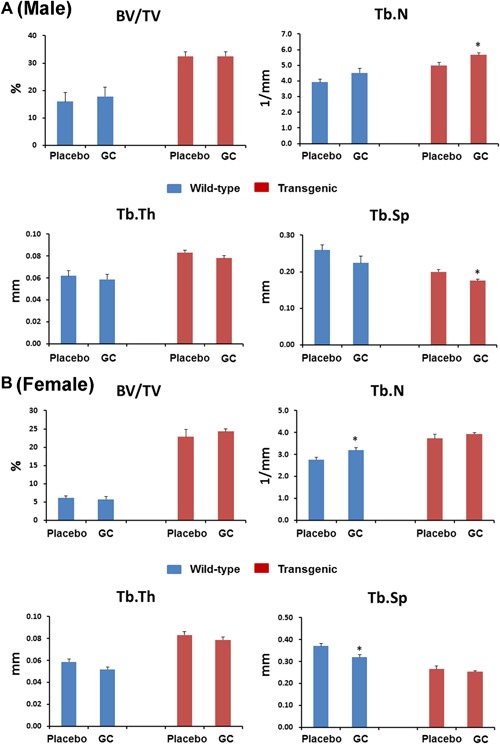
Trabecular bone morphometry measured by micro‐CT. Both male (*A*) and female (*B*) Col2.3‐hWNT16 TG mice treated with placebo displayed significantly higher trabecular BV/TV, Tb.N, Tb.Th, and significantly lower Tb.Sp compared with WT placebo group at 28 days after pellet implantation. GC treatment did not change trabecular BV/TV and Tb.Th in male and female wild‐type and Col2.3‐hWNT16 TG mice compared with placebo‐treated mice. Tb.N and Tb.Sp were also similar in GC and placebo‐treated male wild‐type mice and female transgenic mice, whereas Tb.N was higher in GC‐treated male and female mice compared with placebo‐treated mice. Tb.Sp was lower in GC‐treated male Col2.3‐hWNT16 TG mice and female wild‐type mice compared with genotype‐matched placebo‐treated mice. Values are given as mean ± SEM; *p < 0.05 versus genotype‐matched placebo‐treated mice using Student's *t* test. GC = glucocorticoid.

To further identify the effects of the GC treatment on skeletal properties in wild‐type and Col2.3‐hWNT16 TG mice, we measured bone microarchitectural properties in the distal femur for cancellous bone using μCT. Both male and female Col2.3‐hWNT16 TG mice treated with placebo displayed significantly higher (*p* < 0.001) trabecular BV/TV, Tb.N, and Tb.Th and significantly lower (*p* < 0.001) Tb.Sp compared with WT placebo group at 28 days after pellet implantation (Fig. 3 *A*, *B*). GC treatment did not change trabecular BV/TV and Tb.Th in male and female wild‐type and Col2.3‐hWNT16 TG mice compared with genotype‐matched placebo‐treated mice (Fig. 3 *A*, *B*). Both GC‐ and placebo‐treated wild‐type mice displayed similar values for Tb.N and Tb.Sp in male, whereas Tb.N was significantly higher (*p* < 0.05) and Tb.Sp was significantly lower (*p* < 0.05) in GC‐treated male transgenic mice (Fig. 3 *A*). Tb.N was also significantly higher (*p* < 0.05) and Tb.Sp was significantly lower (*p* < 0.05) in GC‐treated female wild‐type mice compared with placebo‐treated mice (Fig. 3 *B*). Two‐way ANOVA analysis demonstrated no significant interaction effects of GC treatment with genotype for all trabecular μCT measurements (Supplemental Table S2).

### GC treatment significantly compromised cortical bone micro‐architecture at femur midshaft measured by μCT

**Figure 4:**
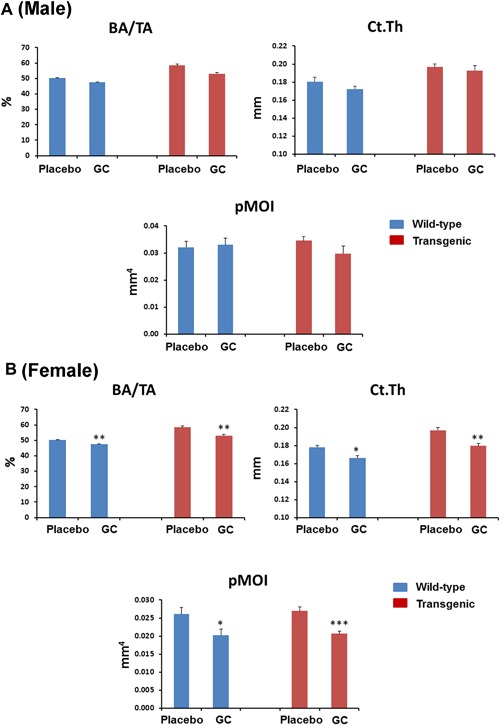
Cortical bone morphometry measured by micro‐CT. Both male (*A*) and female (*B*) Col2.3‐hWNT16 TG mice treated with placebo displayed significantly higher cortical bone area (BA/TA) and thickness (Ct.Th) compared with WT placebo group at 28 days after pellet implantation. GC treatment did not change trabecular BA/TA and Ct.Th in male wild‐type and Col2.3‐hWNT16 TG mice compared with placebo‐treated mice. Both BA/TA and Ct.Th were significantly lower in GC‐treated wild‐type and Col2.3‐hWNT16 TG mice in female compared with placebo‐treated control mice. The polar moment of inertia (pMOI) was also significantly lower in female GC‐treated wild‐type and Col2.3‐hWNT16 TG mice compared with genotype‐matched placebo‐treated mice. Values are given as mean ± SEM; *p < 0.05, **p < 0.005, and ***p < 0.0001 versus genotype‐matched placebo‐treated mice using Student's *t* test. GC = glucocorticoid.

GC treatment did not change cortical BA/TA and Ct.Th in male wild‐type and Col2.3‐hWNT16 TG mice compared with sex‐matched placebo‐treated mice (Fig. 4 *A*). In female mice, both BA/TA and Ct.Th were significantly lower in GC‐treated wild‐type (*p* < 0.005 and *p* < 0.05, respectively) and Col2.3‐hWNT16 TG mice (*p* < 0.005 and *p* < 0.005, respectively) compared with placebo‐treated control mice (Fig. 4 *B*). The polar moment of inertia (pMOI) was also significantly lower in female GC‐treated wild‐type and Col2.3‐hWNT16 TG mice (*p* < 0.05 and *p* < 0.001, respectively) compared with genotype‐matched placebo‐treated mice (Fig. 4 *B*). Two‐way ANOVA analysis demonstrated no significant interaction effects of GC treatment with genotype for all cortical μCT measurements (Supplemental Table S2).

### Biomechanical properties of femurs are compromised due to GC treatment in wild‐type and Col2.3‐hWNT16 TG mice

**Figure 5:**
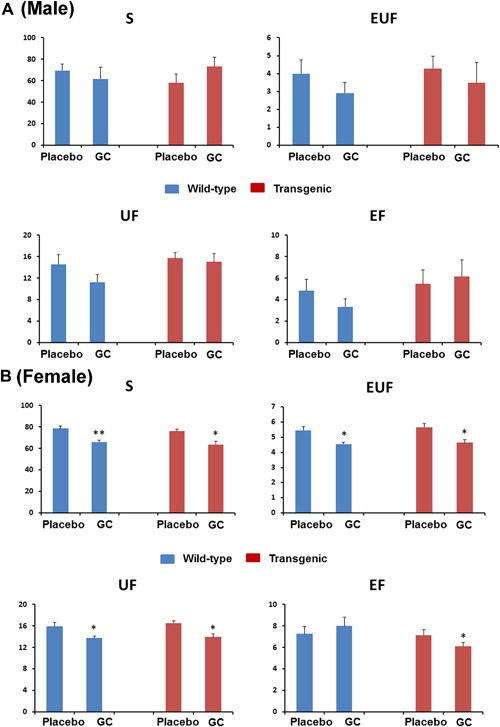
Measurement of bone strength by femur biomechanical test. Female (*B*) wild‐type and Col2.3‐hWNT16 mice treated with GC had significantly lower stiffness, ultimate force, energy to ultimate force, and energy to failure compared with placebo‐treated mice for both genotypes. In contrast, male (*A*) wild‐type and Col2.3‐hWNT16 mice treated with GC had similar values for these biomechanical parameters compared with genotype‐matched placebo‐treated mice. Values are given as mean ± SEM; *p < 0.05 and **p < 0.005 versus genotype‐matched placebo‐treated mice using Student's *t* test. GC = glucocorticoid.

To identify the effect on GC treatment in mechanical properties of the skeleton in wild‐type and Col2.3‐hWNT16 mice, we tested femurs from these mice using monotonic 3‐point bending tests. Female wild‐type mice treated with GC had significantly lower stiffness (*p* < 0.005), ultimate force (*p* < 0.05), and energy to ultimate force (*p* < 0.05) compared with sex‐matched placebo‐treated mice (Fig. 5 *B*). Female Col2.3‐hWNT16 mice treated with GC also displayed significantly lower stiffness (*p* < 0.05), ultimate force (*p* < 0.05), energy to ultimate force (*p* < 0.05), and energy to failure (*p* < 0.05) compared with placebo‐treated Col2.3‐hWNT16 control mice (Fig. 5 *B*). In contrast, male wild‐type mice treated with GC had a trend for lower values and male Col2.3‐hWNT16 mice treated with GC had similar values for these biomechanical parameters compared with genotype‐matched placebo‐treated mice (Fig. 5 *A*). Two‐way ANOVA analysis demonstrated no significant interaction effects of GC treatment with genotype for all femur biomechanical parameters (Supplemental Table S2).

### Serum markers of bone formation are significantly influenced in both wild‐type and Col2.3‐hWNT16 TG mice due to GC treatment

**Figure 6:**
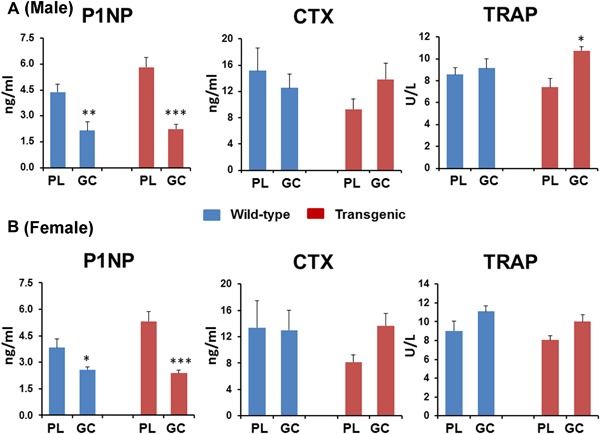
Analyses of serum bone formation and resorption markers. Both wild‐type and Col2.3‐hWNT16 mice showed significantly lower levels of serum P1NP at 2 weeks after pellet implantation due to GC treatment compared with genotype‐matched placebo‐treated mice. Serum levels of CTX and TRAP were similar in GC‐treated wild‐type mice compared with placebo‐treated mice in both male (*A*) and female (*B*) mice at 28 days after pellet implantation. A slightly higher but nonsignificant level of CTX and TRAP were observed in GC‐treated Col2.3‐hWNT16 mice compared with placebo group in both sexes. Values are given as mean ± SEM; *p < 0.05, **p < 0.005, and ***p < 0.0001versus genotype‐matched placebo‐treated mice using Student's *t* test. GC = glucocorticoid.

To understand potential changes in serum biochemistry induced by GC treatment in wild‐type and Col2.3‐hWNT16 mice, we measured biomarkers related to skeletal metabolism in serum samples. At 2 weeks after pellet implantation, both wild‐type and Col2.3‐hWNT16 male mice showed significantly lower levels of serum P1NP (*p* < 0.005 and *p* < 0.001, respectively), a bone formation marker, due to GC treatment compared with genotype‐matched placebo‐treated mice (Fig. 6 *A*). Similarly, female GC‐treated wild‐type and transgenic mice also showed significantly lower levels of serum P1NP (*p* < 0.05 and *p* < 0.001, respectively) compared with genotype‐matched placebo‐treated mice (Fig. 6 *A*). However, the serum level of P1NP was re‐established to the baseline level at 4 weeks post‐treatment (data not shown). At 28 days after pellet implantation, serum levels of CTX and TRAP were similar in GC‐treated wild‐type mice compared with placebo‐treated mice in both male and female (Fig. 6 *B*). A slightly higher but nonsignificant level of CTX was observed in GC‐treated Col2.3‐hWNT16 mice compared with placebo group in both sexes (Fig. 6 *B*). In addition, significantly higher (*p* < 0.05) level of TRAP in male and a slightly higher but nonsignificant level of TRAP were observed in female GC‐treated Col2.3‐hWNT16 mice compared with placebo group (Fig. 6 *B*). Also, similar levels of calcium, phosphorus, and creatinine were detected in GC‐treated wild‐type and Col2.3‐hWNT16 mice compared with their placebo‐treated WT littermates in both male and female (Table 1).

**Table 1:** Serum Biochemistry in Male and Female Wild‐Type and Col2.3‐hWNT16 Transgenic Mice Treated With Placebo and GC

	Male (WT)	Male (TG)	Female (WT)	Female (TG)
Ca (mg/dL)				
Placebo	9.1 ± 0.2	8.7 ± 0.2	9.2 ± 0.1	9.2 ± 0.1
GC	9.5 ± 0.3	9.2 ± 0.1	9.2 ± 0.2	9.1 ± 0.2
*p* value	*0.25*	*0.10*	*0.84*	*0.82*
Phos (mg/dL)				
Placebo	8.1 ± 0.6	7.9 ± 0.6	10.1 ± 1.2	8.7 ± 0.7
GC	9.2 ± 0.4	6.7 ± 0.6	9.6 ± 1.3	8.8 ± 0.9
*p* value	*0.10*	*0.11*	*0.79*	*0.97*
CREA (mg/dL)				
Placebo	0.37 ± 0.02	0.36 ± 0.02	0.41 ± 0.02	0.40 ± 0.01
GC	0.40 ± 0.02	0.38 ± 0.03	0.40 ± 0.02	0.39 ± 0.02
*p* value	0.09	0.50	0.60	*0.77*

WT = wild type; TG = Col2.3‐hWNT16 transgenic; GC = glucocorticoid; Ca = calcium; Phos = phosphorus; CREA = creatinine.

Values are mean ± SEM.

### Lean body mass is significantly decreased in both wild‐type and Col2.3‐hWNT16 TG mice due to GC treatment

**Figure 7:**
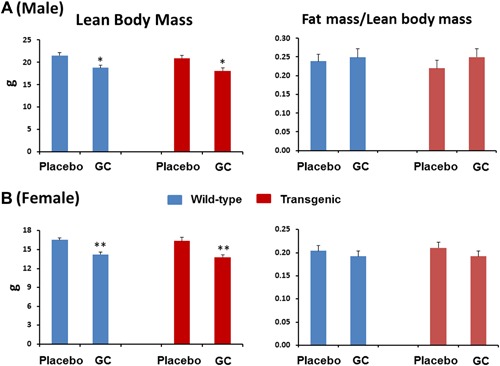
Lean body and fat mass measured by DXA. The lean body mass derived from DXA collected at 28 days after pellet implantation showed that both wild‐type and Col2.3‐hWNT16 mice treated with GC had significantly lower lean body mass in male (*A*) and female (*B*) mice compared with their placebo‐treated WT littermates. In addition, both wild‐type and Col2.3‐hWNT16 mice treated with GC displayed similar fat mass values normalized by the lean body mass compared with their genotype‐matched placebo groups in male and female. Values are given as mean ± SEM; *p < 0.05 and **p < 0.005 versus genotype‐matched placebo‐treated mice using Student's *t* test. GC = glucocorticoid.

We measured lean body mass and fat mass normalized by the lean body mass derived from DXA collected at 28 days after pellet implantation. Wild‐type mice treated with GC showed significantly lower lean body mass in male (*p* < 0.05) and female (*p* < 0.005) compared with the placebo‐treated mice (Fig. 7 *A*, *B*). Similarly, Col2.3‐hWNT16 mice treated with GC showed significantly lower lean body mass in male (*p* < 0.05) and female (*p* < 0.005) compared with their placebo‐treated mice (Fig. 7 *A*, *B*). Both wild‐type and Col2.3‐hWNT16 mice treated with GC showed similar fat mass values compared with their genotype‐matched placebo groups in male and female (Fig. 7 *A*, *B*). Two‐way ANOVA analysis demonstrated no significant interaction effects of GC treatment with genotype for lean body and fat mass (Supplemental Table S2).

### Muscle mass is significantly influenced in both wild‐type and Col2.3‐hWNT16 TG male mice due to GC treatment

**Figure 8:**
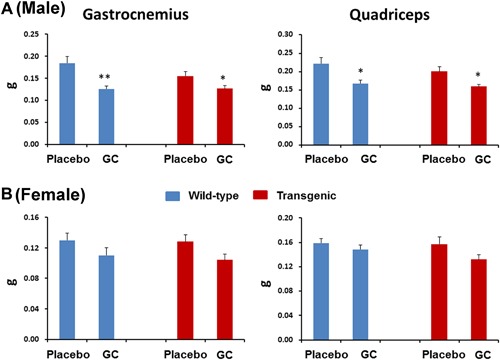
Measurement of muscle mass. Both wild‐type and Col2.3‐hWNT16 mice treated with GC showed significantly lower gastrocnemius and quadriceps muscle mass in male (*A*) compared with their placebo‐treated WT littermates. In addition, a trend of lower gastrocnemius and quadriceps muscle mass in WT and Col2.3‐hWNT16 GC‐treated mice compared with their genotype‐matched placebo groups in female (*B*). Values are given as mean ± SEM; *p < 0.05 and **p < 0.005 versus genotype‐matched placebo‐treated mice using Student's *t* test. GC = glucocorticoid.

Wild‐type mice treated with GC showed significantly lower gastrocnemius (*p* < 0.005) and quadriceps (*p* < 0.05) muscle mass in male compared with the sex‐matched placebo‐treated mice (Fig. 8 *A*). Col2.3‐hWNT16 male mice treated with GC also showed significantly lower gastrocnemius (*p* < 0.05) and quadriceps (*p* < 0.05) muscle mass compared with their placebo‐treated mice (Fig. 8 *A*). In addition, a trend of lower gastrocnemius and quadriceps muscle mass in both WT and Col2.3‐hWNT16 GC‐treated mice compared with their genotype‐matched placebo groups in female (Fig. 8 *B*) was found. Two‐way ANOVA analysis demonstrated no significant interaction effects of GC treatment with genotype for muscle mass (Supplemental Table S2).

### Expression of bone formation and resorption related genes are changed in both wild‐type and Col2.3‐hWNT16 TG mice due to GC treatment

**Figure 9:**
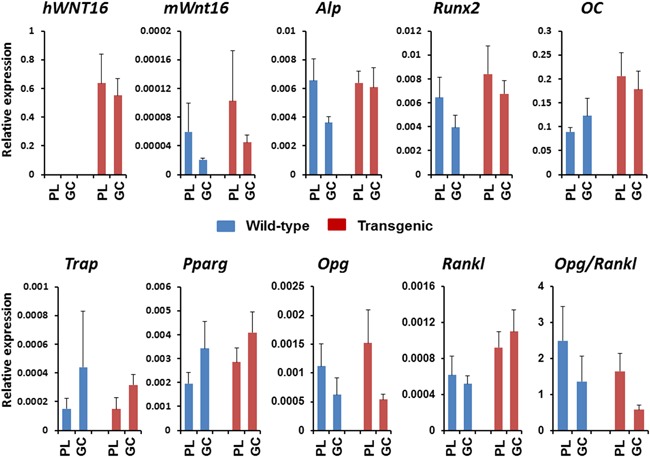
Analyses of gene expression in the bone tissue. A trend of lower levels of *hWNT16* expression was detected in bone tissue (femur) in Col2.3‐hWNT16 mice treated with GC compared with placebo‐treated mice. In addition, the level of endogenous mouse *Wnt16* mRNA expression was lower in both wild‐type and Col2.3‐hWNT16 mice treated with GC compared with their genotype‐matched placebo groups. A trend of lower mRNA levels of *Alp* and *Runx2* were observed in both wild‐type and Col2.3‐hWNT16 mice treated with GC compared with their genotype‐matched placebo groups. The levels of *osteocalcin*, *trap*, and *ctsk* mRNA expressions were similar. In addition, a trend of lower expression of *Opg* and *Opg*/*Rankl* ratio were detected in the GC‐treated wild‐type and transgenic mice compared with their genotype‐matched placebo groups.

We detected a trend of lower levels of *hWNT16* expression in bone tissue (femur) in Col2.3‐hWNT16 mice treated with GC compared with placebo‐treated mice (Fig. 9). In addition, the level of endogenous mouse *Wnt16* mRNA expression was slightly but nonsignificantly lower in both wild‐type and Col2.3‐hWNT16 mice treated with GC compared with their genotype‐matched placebo groups (Fig. 9). A trend of lower mRNA levels of *Alp* in wild‐type and *Runx2* in both wild‐type and Col2.3‐hWNT16 mice were observed treated with GC compared with their genotype‐matched placebo groups (Fig. 9). The levels of *osteocalcin* and *Trap* mRNA expressions were similar in both placebo‐ and GC‐treated mice for both genotypes (Fig. 9). Further, a higher but nonsignificant level of *Pparg* mRNA expression was observed in both placebo‐ and GC‐treated mice for both genotypes (Fig. 9). In addition, trends of lower expression of Opg and Opg/Rankl ratio were detected in the GC‐treated wild‐type and transgenic mice compared with their genotype‐matched placebo groups (Fig. 9).

## Discussion

Our data demonstrate that GC had significant detrimental effect on lean body mass and muscle mass in both male and female wild‐type and Col2.3‐hWNT16 transgenic mice. GC treatment also significantly lowered the femoral aBMD and BMC in female mice for both genotypes. Micro‐CT analysis revealed that GC significantly decreased cortical bone area and thickness in female mice, which correlated with the reduction of bone strength in these mice for both genotypes. In contrast, the trabecular bone parameters in femur and spine were not significantly changed by GC treatment in male and female wild‐type and Col2.3‐hWNT16 transgenic mice. Serum biomarkers showed that P1NP levels were significantly reduced in both male and female wild‐type and transgenic mice. We also observed a trend of lower *mWnt16*, *Runx2*, *Opg*, and *Opg/Rankl* ratio in GC‐treated mice for both genotypes. Together these results suggest that although overexpression of WNT16 increased bone mass significantly in both vehicle and GC‐treated mice, it did not completely prevent bone loss in mice due to glucocorticoid treatment.

In this study, we observed sex‐ and site‐specific influence of GC on bone density and mineral content due to glucocorticoid treatment. Although whole body and lumbar spine aBMD and BMC were not significantly changed due to GC treatment in male and female wild‐type and Col2.3‐hWNT16 transgenic mice (Supplemental Figs. S1 and S2), GC‐treated female mice showed significantly lowered aBMD and BMC in femur for both genotypes compared with placebo‐treated mice (Fig. 2 *B*). Male wild‐type and transgenic mice treated with GC had slightly but nonsignificantly lower femoral BMC compared with the placebo‐treated mice (Fig. 2 *A*). Previously, several studies demonstrated that GC influence both cortical and trabecular bone density and microarchitecture involving both axial and appendicular skeletons.^14^, ^17^, ^18^, ^19^ Although some studies found lower cortical and trabecular bone density due to GC treatment,^17^, ^18^, ^19^ other studies showed trabecular bone volume over tissue volume was not significantly changed or even increased.^14^, ^20^ Most of these studies demonstrated that cortical bone volume and thickness were significantly lowered by GC treatment.^14^, ^18^, ^19^ Consistent with these studies, we also discovered femur cortical bone area and thickness were significantly decreased due to GC treatment in both wild‐type and transgenic mice (Fig. 4 *B*). In contrast, trabecular bone microarchitecture parameters were not changed due to GC treatment in both distal femur and spine. These could be attributable to the different doses and duration of the GC used as well as genetic backgrounds of mice employed in different studies. Importantly, the predominant cortical effect in female mice for both genotypes led to significantly lowered bone biomechanical parameters due to GC treatment (Fig. 5 *B*).

Previous studies demonstrated that administration of GC led to initial increase in biochemical markers of bone resorption followed by a suppression of both bone formation and resorption markers.^3^, ^4^ Significantly lower serum levels of P1NP (Fig. 6) in GC‐treated wild‐type and transgenic mice as early as 2 weeks post‐treatment in the current study suggest that the detrimental effect of GC on bone formation could be a major factor for decreased bone mineral density and strength in these mice, and higher level of WNT16 expression in the transgenic mice offers limited protection to the negative effects on bone formation. In addition, the serum level of P1NP was re‐established to the baseline level at 4 weeks post‐treatment (data not shown), suggesting that suppression of bone formation marker is temporary. Although serum levels of CTX and TRAcP5b did not change significantly between placebo and GC treatment in the wild‐type mice in both male and female, a trend of higher levels of CTX and TRAcP5b were observed in the GC‐treated transgenic mice in both sexes (Fig. 6), suggesting higher WNT16 expression in the transgenic mice offers no protection on bone resorption. GC treatment might prolong osteoclast survival in the transgenic mice, which is evident by higher TRAP level in this genotype.

Although GC affects both bone formation and resorption, the major or primary effect is the decrease of bone formation due to the direct effect of GC on cells of the osteoblastic lineage.^5^, ^21^, ^22^, ^23^ GC decreases the proliferation of osteoblast as well as impairs osteoblastic differentiation and function.^5^, ^21^ This is due to inhibition of key regulators of osteoblastogenesis or repression of genes important in osteoblast differentiation. In addition, GC redirects the stromal cells toward cells of other lineages such as adipocyte as well as increases early death of osteoblasts by increasing the activation of apoptotic genes.^21^ In this study, we observed a trend of lower expression of *Alp*, *Runx2*, *Opg*, and *Opg*/*Rankl* ratio and higher expression of *Pparg* in the GC‐treated wild‐type and transgenic mice compared with their genotype‐matched placebo groups (Fig. 9). In addition, endogenous mouse *Wnt16* mRNA expression was lower and a trend of lower levels of *hWNT16* expression in GC‐treated mice for both genotypes. Decreased expression of bone formation genes and inhibition of Wnt‐signaling pathway that is critical for bone formation and differentiation along with limited protection on bone formation due to higher hWNT16 expression all together had a tremendous negative role on skeletal homeostasis in GC‐treated mice.

Previously, several approaches were undertaken to prevent GC‐induced bone loss using different interventions. For example, therapies involving intermittent PTH,^22^, ^24^ sost‐antibody,^18^ anti‐Rankl antibody,^19^ IL‐6 neutralizing antibody,^25^ as well as inhibition of HSP90^26^ and endoplasmic reticulum stress^23^ prevented the bone phenotypes due to GC treatment to variable degrees in vivo. Moreover, genetic approaches using knockout mice of mitogen‐activated protein kinase phosphatase 1,^27^ plasminogen activator inhibitor 1,^28^ Npy,^29^ sost,^14^ and autophagy‐related gene 7^30^ have been used to prevent GC‐induced bone loss but resulted in variable outcomes. Our transgenic (TG) mice that overexpress human WNT16 in bone tissue displayed significantly higher bone mineral density in whole body, hip, and spine.^13^ They also showed significantly higher trabecular bone volume and cortical bone area and thickness as well as higher bone strength. WNT16 overexpression had a dramatic positive influence on bone formation but minimum effect on bone resorption. Because one of the main effects of GC is the decrease of bone formation due to the direct effect on cells of the osteoblastic lineage, WNT16 overexpression could have the potential to prevent bone loss and prevent bone fragility due to glucocorticoid treatment in mice. However, our data in the current study shows that WNT16 overexpression in the transgenic mice does not prevent bone loss. It should be noted that WNT16‐TG mice treated with GC had higher BMD and strength than WT mice. Previous studies demonstrated that GC‐mediated bone loss involves Wnt‐signaling pathway, particularly Wnt‐antagonists such as Sost and Dkk1.^14^, ^15^, ^18^ Ohnaka and colleagues showed that GC suppressed Wnt3a in cultured human osteoblasts;^31^ however, it is yet to be determined whether any specific Wnt molecule/s play a critical role in this process in vivo. Our current study demonstrates that GC‐induced loss of bone might be less dependent on the Wnt16‐signaling pathway and mainly regulated by other molecules/factors.

GC‐induced myopathy was observed in other animal studies and in patients,^2^, ^14^, ^32^ which involves proteolysis of muscle fibers due to activation of lysosomal and ubiquitin‐proteasome enzymes or by upregulation of myostatin, a negative regulator of muscle mass. We also observed that GC had a significant detrimental effect on lean body mass and muscle mass in both male and female wild‐type and Col2.3‐hWNT16 transgenic mice (Fig. 8), suggesting higher WNT16 expression in the transgenic mice offers no protection on muscle loss. These results also suggest that loss of muscle mass might be independent on the Wnt16‐signaling pathway and predominantly regulated by other factors.

The dose of glucocorticoid (2.1 mg/kg body weight) selected in this study had been previously shown to produce glucocorticoid‐induced osteoporosis in mouse model.^5^, ^14^ This dose is approximately two to three times higher than the high therapeutic dose (40 to 60 mg/d in 60 kg individual) used in human. The dose necessary to produce a similar effect to clinically used dose in humans is consistently higher in mice due to higher metabolic rate and higher clearance than in humans. Also, we used adult mice to avoid the difference on bone phenotypes during the growth period. The duration of 28 days is equivalent to approximately 2 years in humans, representing long‐term effects or chronic GC administration to humans.

Some of the limitations of our studies include: 1) we used 6 animals per group; 2) we tested only one dose of GC; 3) we did not investigate gene expression studies in male mice; 4) we did not analyze bone histomorphometry; and 5) we did not investigate thoroughly the mechanism of muscle loss in our mouse model.

In conclusion, we demonstrated that GC had a significant detrimental effect on lean body mass and muscle mass in both male and female wild‐type and WNT16‐transgenic mice. GC treatment also significantly lowered the femoral aBMD and BMC, cortical bone area and thickness, and reduction of bone strength in female mice. Serum biomarkers showed that P1NP levels were significantly reduced in both male and female wild‐type and WNT16‐transgenic mice. We also observed a trend of lower *Runx2*, *Opg*, and *Opg/Rankl* ratio in GC‐treated mice for both genotypes. Together these results suggest that overexpression of WNT16 was insufficient to prevent bone loss in mice due to glucocorticoid treatment, although WNT16‐TG mice treated with GC had higher BMD and strength than WT mice.

## Disclosures

All authors state that they have no conflicts of interest.

## Supporting information

Supporting Figure S1.

Supporting Table S1.

Supporting Table S2.

## References

[1] Overman RA , Yeh JY , Deal CL. Prevalence of oral glucocorticoid usage in the United States: a general population perspective. Arthritis Care Res (Hoboken). 2013; 65 (2):294–8. 22807233 10.1002/acr.21796

[2] Mirza F , Canalis E. Management of endocrine disease: secondary osteoporosis: pathophysiology and management. Eur J Endocrinol. 2015; 173 (3):R131–51. 25971649 10.1530/EJE-15-0118 PMC4534332

[3] Frenkel B , White W , Tuckermann J. Glucocorticoid‐induced osteoporosis. Adv Exp Med Biol. 2015; 872:179–215. 26215995 10.1007/978-1-4939-2895-8_8 PMC5905346

[4] Warriner AH , Saag KG. Glucocorticoid‐related bone changes from endogenous or exogenous glucocorticoids. Curr Opin Endocrinol Diabetes Obes. 2013; 20 (6):510–6. 24468753 10.1097/01.med.0000436249.84273.7b

[5] O'Brien CA , Jia D , Plotkin LI , et al. Glucocorticoids act directly on osteoblasts and osteocytes to induce their apoptosis and reduce bone formation and strength. Endocrinology. 2004; 145 (4):1835–41. 14691012 10.1210/en.2003-0990

[6] Kanis JA , Johansson H , Oden A , et al. A meta‐analysis of prior corticosteroid use and fracture risk. J Bone Miner Res. 2004; 19 (6):893–9. 15125788 10.1359/JBMR.040134

[7] Hermann AP , Abrahamsen B. The bisphosphonates: risks and benefits of long term use. Curr Opin Pharmacol. 2013; 13 (3):435–9. 23434194 10.1016/j.coph.2013.02.002

[8] Lems WF , Saag K. Bisphosphonates and glucocorticoid‐induced osteoporosis: cons. Endocrine. 2015; 49 (3):628–34. 26041376 10.1007/s12020-015-0639-1 PMC4512570

[9] Baron R , Kneissel M. WNT signaling in bone homeostasis and disease: from human mutations to treatments. Nat Med. 2013; 19 (2):179–92. 23389618 10.1038/nm.3074

[10] Zheng HF , Tobias JH , Duncan E , et al. WNT16 influences bone mineral density, cortical bone thickness, bone strength, and osteoporotic fracture risk. PLoS Genet. 2012; 8 (7):e1002745. 22792071 10.1371/journal.pgen.1002745 PMC3390364

[11] Koller DL , Zheng HF , Karasik D , et al. Meta‐analysis of genome‐wide studies identifies WNT16 and ESR1 SNPs associated with bone mineral density in premenopausal women. J Bone Miner Res. 2013; 28 (3):547–58. 23074152 10.1002/jbmr.1796 PMC3691010

[12] Movérare‐Skrtic S , Henning P , Liu X , et al. Osteoblast‐derived WNT16 represses osteoclastogenesis and prevents cortical bone fragility fractures. Nat Med. 2014; 20(11):1279–88. 25306233 10.1038/nm.3654 PMC4392888

[13] Alam I , Alkhouli M , Gerard‐O'Riley RL , et al. Osteoblast‐specific overexpression of human WNT16 increases both cortical and trabecular bone mass and structure in mice. Endocrinology. 2016; 157 (2):722–36. 26584014 10.1210/en.2015-1281 PMC4733115

[14] Sato AY , Cregor M , Delgado‐Calle J , et al. Protection From glucocorticoid‐induced osteoporosis by anti‐catabolic signaling in the absence of Sost/Sclerostin. J Bone Miner Res. 2016; 31 (10):1791–802. 27163932 10.1002/jbmr.2869 PMC8499032

[15] Guañabens N , Gifre L , Peris P. The role of Wnt signaling and sclerostin in the pathogenesis of glucocorticoid‐induced osteoporosis. Curr Osteoporos Rep. 2014; 12(1):90–7. 24488619 10.1007/s11914-014-0197-0

[16] Bouxsein ML , Boyd SK , Christiansen BA , Guldberg RE , Jepsen KJ , Müller R. Guidelines for assessment of bone microstructure in rodents using micro‐computed tomography. J Bone Miner Res. 2010; 25 (7):1468–86. 20533309 10.1002/jbmr.141

[17] Lane NE , Yao W , Balooch M , et al. Glucocorticoid‐treated mice have localized changes in trabecular bone material properties and osteocyte lacunar size that are not observed in placebo‐treated or estrogen‐deficient mice. J Bone Miner Res. 2006; 21(3):466–76. 16491295 10.1359/JBMR.051103 PMC1797152

[18] Yao W , Dai W , Jiang L , et al. Sclerostin‐antibody treatment of glucocorticoid‐induced osteoporosis maintained bone mass and strength. Osteoporos Int. 2016; 27 (1):283–94. 26384674 10.1007/s00198-015-3308-6 PMC4958115

[19] Hofbauer LC , Zeitz U , Schoppet M , et al. Prevention of glucocorticoid‐induced bone loss in mice by inhibition of RANKL. Arthritis Rheum. 2009; 60 (5):1427–37. 19404943 10.1002/art.24445

[20] Grahnemo L , Jochems C , Andersson A , et al. Possible role of lymphocytes in glucocorticoid‐induced increase in trabecular bone mineral density. J Endocrinol. 2015; 224 (1):97–108. 25359897 10.1530/JOE-14-0508 PMC4254076

[21] Yao W , Cheng Z , Busse C , Pham A , Nakamura MC , Lane NE. Glucocorticoid excess in mice results in early activation of osteoclastogenesis and adipogenesis and prolonged suppression of osteogenesis: a longitudinal study of gene expression in bone tissue from glucocorticoid‐treated mice. Arthritis Rheum. 2008; 58(6):1674–86. 18512788 10.1002/art.23454 PMC3892702

[22] Weinstein RS , Jilka RL , Almeida M , Roberson PK , Manolagas SC. Intermittent parathyroid hormone administration counteracts the adverse effects of glucocorticoids on osteoblast and osteocyte viability, bone formation, and strength in mice. Endocrinology. 2010; 151 (6):2641–9. 20410195 10.1210/en.2009-1488 PMC2875832

[23] Sato AY , Tu X , McAndrews KA , Plotkin LI , Bellido T. Prevention of glucocorticoid induced‐apoptosis of osteoblasts and osteocytes by protecting against endoplasmic reticulum (ER) stress in vitro and in vivo in female mice. Bone. 2015; 73:60–8. 25532480 10.1016/j.bone.2014.12.012 PMC4336847

[24] Postnov A , De Schutter T , Sijbers J , Karperien M , De Clerck N. Glucocorticoid‐induced osteoporosis in growing mice is not prevented by simultaneous intermittent PTH treatment. Calcif Tissue Int. 2009; 85 (6):530–7. 19844646 10.1007/s00223-009-9301-3

[25] Li X , Zhou ZY , Zhang YY , Yang HL. IL‐6 contributes to the defective osteogenesis of bone marrow stromal cells from the vertebral body of the glucocorticoid‐induced osteoporotic mouse. PLoS One. 2016; 11 (4):e0154677. 27128729 10.1371/journal.pone.0154677 PMC4851291

[26] Chen H , Xing J , Hu X , et al. Inhibition of heat shock protein 90 rescues glucocorticoid‐induced bone loss through enhancing bone formation. J Steroid Biochem Mol Biol. 2017; 171:236–46. 28408351 10.1016/j.jsbmb.2017.04.004

[27] Conradie MM , Cato AC , Ferris WF , de Wet H , Horsch K , Hough S. MKP‐1 knockout does not prevent glucocorticoid‐induced bone disease in mice. Calcif Tissue Int. 2011; 89 (3):221–7. 21698455 10.1007/s00223-011-9509-x

[28] Tamura Y , Kawao N , Yano M , et al. Role of plasminogen activator inhibitor‐1 in glucocorticoid‐induced diabetes and osteopenia in mice. Diabetes. 2015; 64 (6):2194–206. 25552599 10.2337/db14-1192

[29] Wang FS , Lian WS , Weng WT , et al. Neuropeptide Y mediates glucocorticoid‐induced osteoporosis and marrow adiposity in mice. Osteoporos Int. 2016; 27 (9):2777–89. 27080706 10.1007/s00198-016-3598-3

[30] Lin NY , Chen CW , Kagwiria R , et al. Inactivation of autophagy ameliorates glucocorticoid‐induced and ovariectomy‐induced bone loss. Ann Rheum Dis. 2016; 75 (6):1203–10. 26113650 10.1136/annrheumdis-2015-207240

[31] Ohnaka K , Tanabe M , Kawate H , Nawata H , Takayanagi R. Glucocorticoid suppresses the canonical Wnt signal in cultured human osteoblasts. Biochem Biophys Res Commun. 2005; 329 (1):177–81. 15721290 10.1016/j.bbrc.2005.01.117

[32] Sato AY , Richardson D , Cregor M , et al. Glucocorticoids induce bone and muscle atrophy by tissue‐specific mechanisms upstream of E3 ubiquitin ligases. Endocrinology. 2017; 158(3):664–77. 28359087 10.1210/en.2016-1779 PMC5460781

