# Supplementary material for: Overexpression of WNT16 Does Not Prevent Cortical Bone Loss Due to Glucocorticoid Treatment in Mice
Source: JBMR Plus. 2018 Oct 23;3(4):e10084. doi: 10.1002/jbm4.10084 (PMC6478588; doi:10.1002/jbm4.10084)
Supplement: Supplementary file 1 — Supporting Figure S1. [file JBM4-3-na-s001.docx]

**
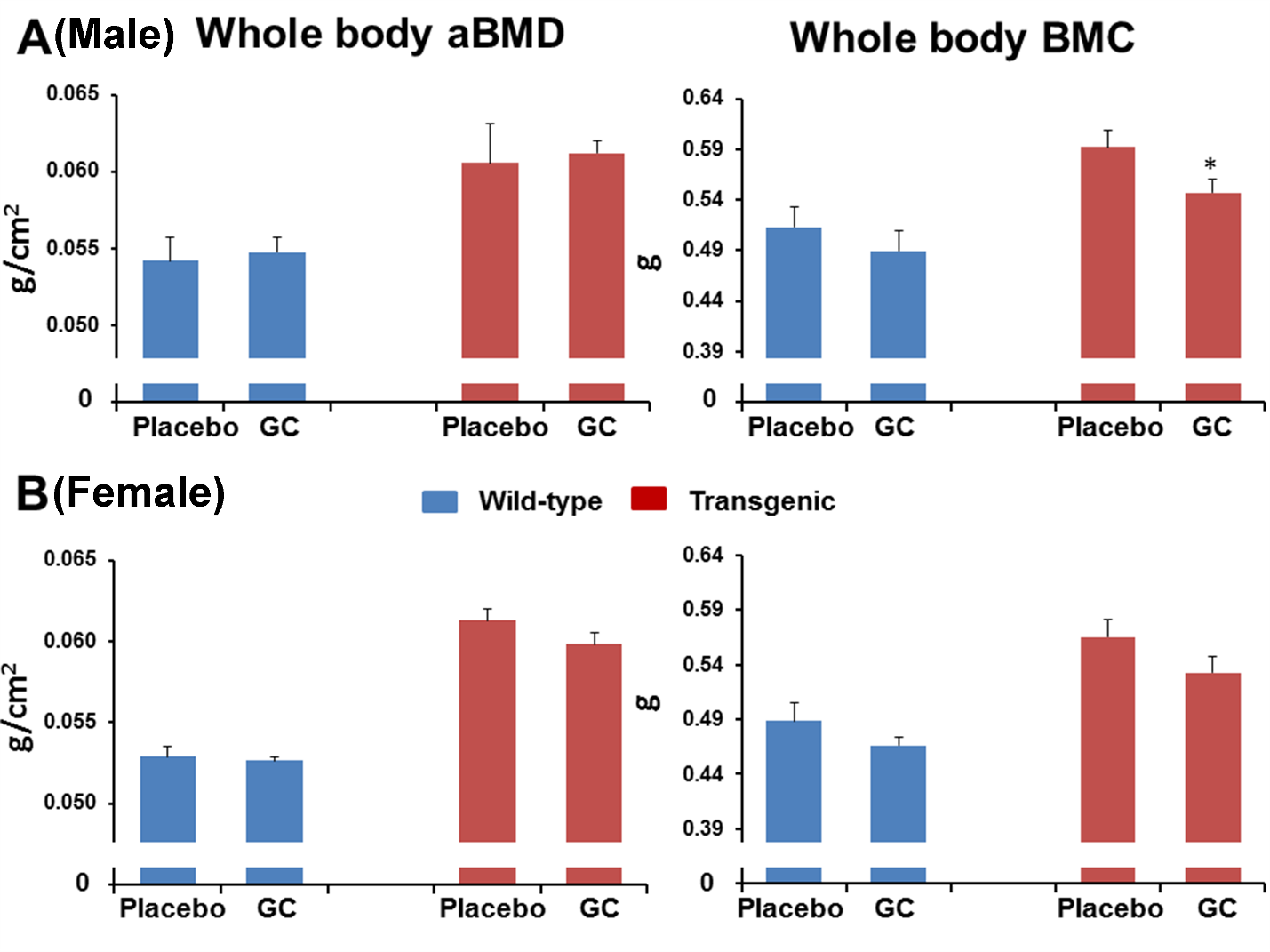
Supplemental Fig. S1.** Whole body aBMD and BMC measured by DXA. Male and female Col2.3-hWNT16 TG mice treated with placebo had significantly higher whole body aBMD and BMC compared to WT placebo group at 28-days after pellet implantation. GC treatment did not significantly change whole body aBMD in male and female wild-type and Col2.3-hWNT16 TG mice compared to placebo-treated mice. Although a slightly lower whole body BMC was observed in GC-treated male and female mice in both genotypes, it did not reach to the significant level except in male. In addition, GC treatment did not significantly change spine aBMD and BMC in male and female wild-type and Col2.3-hWNT16 TG mice compared to placebo-treated mice. Values are given as mean ± SEM *p<0.05 vs. genotype-matched placebo treated mice using Student’s t test. GC: glucocorticoid

**
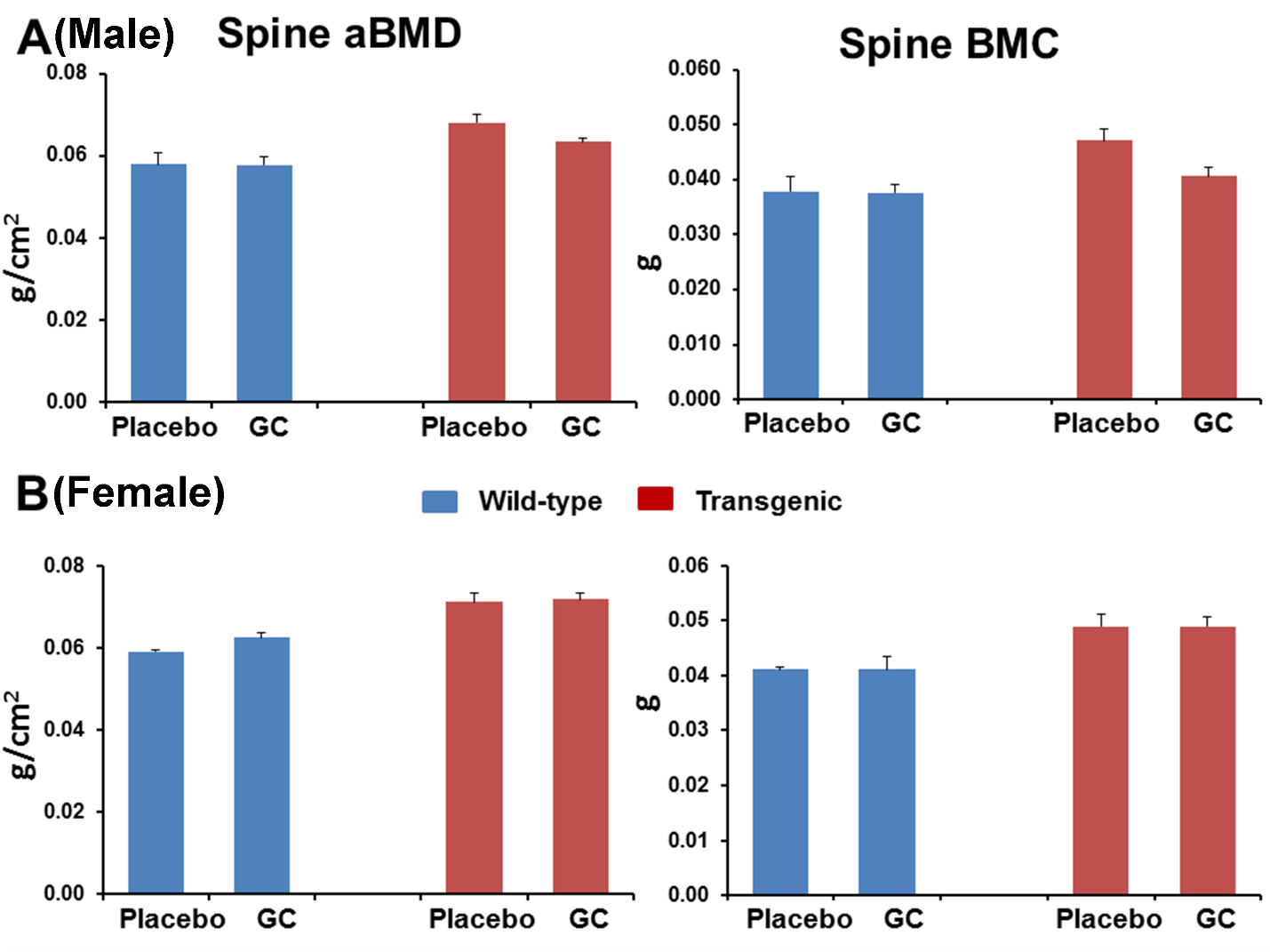
**

**Supplemental Fig. S2.** Lumbar spine (L1-5) aBMD and BMC measured by DXA. Male and female Col2.3-hWNT16 TG mice treated with placebo had significantly higher spine (L1-5) aBMD and BMC compared to WT placebo group at 28-days after pellet implantation. In addition, GC treatment did not significantly change spine aBMD and BMC in male and female wild-type and Col2.3-hWNT16 TG mice compared to placebo-treated mice.
