# Supplementary material for: Overexpression of WNT16 Does Not Prevent Cortical Bone Loss Due to Glucocorticoid Treatment in Mice
Source: JBMR Plus. 2018 Oct 23;3(4):e10084. doi: 10.1002/jbm4.10084 (PMC6478588; doi:10.1002/jbm4.10084)
Supplement: Supplementary file 2 [file JBM4-3-na-s002.docx]

Supplemental Table 1. Baseline body weight and DXA values in male and female wild-type and transgenic mice treated with placebo and GC[a]

		Male (WT)	Male (TG)	Female (WT)	Female (TG)
Body weight (g)	Placebo	27.7 ± 0.8	27.2 ± 0.6	22.0 ± 0.5	21.3 ± 0.9
	GC	27.1 ± 0.7	27.3 ± 0.4	21.8 ± 0.5	21.1 ± 0.4
	p-value	*0.56*	*0.88*	*0.84*	*0.79*
Whole body aBMD (g/cm^2)	Placebo	0.0534 ± 0.002	0.0587 ± 0.002	0.0510 ± 0.001	0.0589 ± 0.001
	GC	0.0547 ± 0.002	0.0610 ± 0.001	0.0506 ± 0.001	0.0591 ± 0.001
	p-value	*0.64*	*0.2*	*0.72*	*0.86*
Whole body BMC (g)	Placebo	0.496 ± 0.02	0.558 ± 0.02	0.439 ± 0.02	0.515 ± 0.02
	GC	0.503 ± 0.03	0.569 ± 0.02	0.432 ± 0.01	0.518 ± 0.01
	p-value	*0.84*	*0.7*	*0.74*	*0.87*
Femur aBMD (g/cm^2)	Placebo	0.0832 ± 0.005	0.0943 ± 0.004	0.0694 ± 0.001	0.0833 ± 0.002
	GC	0.0842 ± 0.006	0.0977 ± 0.004	0.0696 ± 0.001	0.0847 ± 0.001
	p-value	*0.89*	*0.54*	*0.87*	*0.47*
Femur BMC (g)	Placebo	0.0326 ± 0.002	0.0376 ± 0.001	0.0279 ± 0.002	0.0324 ± 0.001
	GC	0.0336 ± 0.002	0.0389 ± 0.001	0.0279 ± 0.001	0.0328 ± 0.001
	p-value	*0.73*	*0.41*	*0.98*	*0.79*
Spine aBMD (g/cm^2)	Placebo	0.0622 ± 0.003	0.0685 ± 0.003	0.0585 ± 0.001	0.0691 ± 0.002
	GC	0.0602 ± 0.004	0.0683 ± 0.002	0.0584 ± 0.001	0.0696 ± 0.002
	p-value	*0.69*	*0.94*	*0.95*	*0.87*
Spine BMC (g)	Placebo	0.0401 ± 0.002	0.0453 ± 0.002	0.0397 ± 0.002	0.0466 ± 0.002
	GC	0.0388 ± 0.003	0.0430 ± 0.002	0.0373 ± 0.001	0.0473 ± 0.002
	p-value	*0.74*	*0.41*	*0.25*	*0.81*

a Values are mean ± SEM

WT: wild-type; TG: Col2.3-hWNT16 transgenic; GC: glucocorticoid; aBMD: area bone mineral density; BMC: bone mineral content
