# Supplementary material for: Overexpression of WNT16 Does Not Prevent Cortical Bone Loss Due to Glucocorticoid Treatment in Mice
Source: JBMR Plus. 2018 Oct 23;3(4):e10084. doi: 10.1002/jbm4.10084 (PMC6478588; doi:10.1002/jbm4.10084)
Supplement: Supplementary file 3 [file JBM4-3-na-s003.docx]

Supplemental Table 2. Two-way ANOVA (interaction effects of GC treatment with genotypes) derived p-values for bone and other phenotypes in male, female and all mice

	All samples	Male	Female
BV/TV (%)	0.57	0.32	0.44
Tb.N (1/mm)	0.60	0.85	0.39
Tb.Th (mm)	0.61	0.42	0.64
Tb.Sp (mm)	0.34	0.46	0.09
BA/TA (%)	0.51	0.72	0.12
Ct.Th (mm)	0.81	0.62	0.32
pMOI (mm^4)	0.33	0.23	0.87
UF (N)	0.55	0.44	0.71
S (N/mm)	0.27	0.26	0.99
EUF (mJ)	0.58	0.86	0.77
EF (mJ)	0.76	0.40	0.18
Femur aBMD (g/cm^2)	0.61	0.86	0.49
Femur BMC (g)	0.67	0.77	0.36
Whole body aBMD (g/cm^2)	0.85	0.79	0.42
Whole body BMC (g)	0.64	0.78	0.76
Spine aBMD (g/cm^2)	0.41	0.33	0.41
Spine BMC (g)	0.49	0.17	0.99
Bone length (mm)	0.47	0.74	0.68
Fat mass (g)	0.92	0.72	0.66
Lean body mass (g)	0.77	0.96	0.79
Fat mass/Lean body mass	0.95	0.66	0.81
Gastrocnemius (g)	0.37	0.20	0.83
Quadriceps (g)	0.95	0.62	0.43

GC: glucocorticoid; aBMD: area bone mineral density; BMC: bone mineral content: BV/TV: bone volume/tissue volume; Tb.N: trabecular number; Tb.th: trabecular thickness; Tb.Sp: trabecular separation; BA/TA: bone area/total area; Ct.Th: cortical thickness; pMOI: polar moment of inertia; UF: ultimate force; S: stiffness; EUF: energy to ultimate force; EF: energy to failure
